# Creating Systems-Level Change to Better Support Expectant and Parenting Young People: A Case Study

**DOI:** 10.1007/s10995-020-02991-7

**Published:** 2020-09-05

**Authors:** Amanda Purington, Erica Stupp, Divine Sebuharara, Brian Maley, Sara Birnel Henderson, Jane Powers

**Affiliations:** 1 Bronfenbrenner Center for Translational Research, Cornell University, 35 Thornwood Drive Suite 200, Ithaca, NY 14853 USA; 2 Bureau of Women, Infant & Adolescent Health, New York State Department of Health, Albany, NY USA; 3 Bronfenbrenner Center for Translational Research, Cornell University, Ithaca, NY USA; 4 Present Address: State University of New York at Binghamton, Binghamton, NY USA; 5 Present Address: Planned Parenthood Federation of America, New York, NY USA

**Keywords:** Case study, Systems-building approach, Collaboration, Young parents, School-based

## Abstract

**Introduction:**

Expectant and parenting young people (young parents) require diverse services to support their health, educational success, and family functioning. Rarely can the needs of young parents be met by a single school or service provider. This case study examines how one large school district funded through the pathways to success initiative was able to facilitate systems change to increase young parents’ access to and use of supportive services.

**Methods:**

Data sources include a needs and resources assessment, quarterly reports documenting grantee effort, sustainability plans, social network analysis, and capstone interviews. All data sources were systematically reviewed to identify the existing context prior to the start of the initiative, the changes that resulted from the initiative, and efforts that could potentially be maintained beyond the grant period.

**Results:**

The community context prior to Pathways implementation was one of disconnected services and missed opportunities. The full-time program coordinator hired by the district focused on systems-level change and facilitated connections between organizations. This greater connectivity contributed to increased collaboration with the goal of producing lasting benefits for young parents.

**Discussion:**

Promoting sustainable connections and collaboration at the systems level can help dismantle barriers to service access and benefit young parents.

## Significance

To bring about broader, sustainable population impact, increased focus has been placed on systems-level interventions, which aim to impact how different services and resources interact to achieve common goals. This article presents a case study of a systems-level change approach implemented within a school district collaborating with partners to better support expectant and parenting students to achieve their academic and parenting goals. The lessons presented could inform other communities seeking to improve collaboration among service providers as well as young parents’ access to and utilization of services.

## Introduction

Expectant and parenting young people (young parents) and their children are at risk for poor developmental, health, educational, and social outcomes; they often require diverse services to achieve educational success and support their families (Lachance et al. 2012; Pinzon and Jones 2012; Savio Beers and Hollo 2009). Rarely can the needs of young parents be met by a single school or service provider; rather, young parents must navigate a challenging, disjointed, and “siloed” system (Lachance et al. 2012). For this reason, systems-level or collaborative approaches have been employed to support pregnant and parenting teens (Radcliff et al. 2018) and, similarly, to improve teen pregnancy prevention efforts (Cassell et al. 2005; Chervin et al. 2005; Kegler et al. 2005; Mueller et al. 2017). A systems-level approach to service delivery identifies opportunities for improvement by considering not only the individual service providers but also the interactions and connections between these providers and how they function as a whole (Emshoff et al. 2007; Foster-Fishman et al. 2007; Leischow and Milstein 2006; Mabry et al. 2008). Resource sharing, increased community awareness and support, development of new resources or programs to meet identified gaps, reduced duplication of services, and policy changes to increase access to services are common outcomes of efforts to improve collaboration (Chervin et al. 2005; Kegler et al. 2005; Mueller et al. 2017). Initiatives that use this approach may then sustain certain efforts beyond the funding period by institutionalizing systems-level changes through policies and procedures within organizations or among community partners (Wolff 2010).

Pathways to success (Pathways), a New York State (NYS) initiative funded by the federal Office of Population Affairs Pregnancy Assistance Fund, sought to support young parents and their families largely through a systems-level approach. The NYS Department of Health (NYSDOH) coordinated the project in collaboration with Cornell University’s Assets Coming Together for Youth Center for Community Action (ACT). Designed to create and improve supportive systems that promote academic achievement and health for young families, Pathways was composed of funded partnerships between a local school district and a community college (funded organizations) in three NYS communities. Pathways aimed to create a sustainable infrastructure of support for young parents and facilitate individual-level outcomes by (1) strengthening organizational and community systems, (2) improving the health and well-being of young families, (3) improving self-sufficiency and educational outcomes for young parents, and (4) increasing awareness of resources to young parents and the organizations serving them. Each funded organization hired a program coordinator who was responsible for implementation. To accomplish initiative goals at the individual level, coordinators conducted comprehensive asset and risk assessments with young parents, which in turn led to a set of referrals tailored to each individual’s needs. However, for young people in search of services, referrals are only the beginning of the process; young people must then enter a system that has, to a large extent, evolved piecemeal rather than being intentionally designed to accommodate them. Funded organizations were expected to exact systems-level change by conducting assessments of community needs and resources, developing plans to address service gaps, building and improving partnerships, and raising awareness among service providers of existing resources for young parents.

Early in the implementation of the Pathways initiative, the efforts of one funded organization stood out as a potentially valuable example of the work required to bring about systems-level change. With the initiative’s dual charge of individual- and systems-level activities, most funded organizations focused more efforts on the former, whereas initial progress reports indicated that this organization, a large school district, focused on the latter. The school district serves a racially, ethnically, and socioeconomically diverse student body; an estimated 630 female high school students in this district give birth each year. This large district operates through a complex administrative structure in which programs have separate funding and oversight, often functioning in isolation. By looking more closely into this organization’s efforts, we sought to better understand the challenges of and approaches to systems-level change. In this paper, we present the results of that closer examination, a case study of one funded organization’s efforts to change service coordination and collaboration within the larger context of the Pathways initiative.

We used a case study approach to delineate (1) the organizational context prior to the initiative, (2) how key players within the school district took action to bring about systems-level changes, and (3) how useful changes might be sustained. Lessons learned from this experience can inform efforts to serve young parents and their families in other school districts and communities.

## Methods

### Data Sources

Several data sources were used. Project planning and implementation data, collected routinely, included findings from a comprehensive needs and resources assessment, quarterly progress reports, sustainability planning documentation, and a social network analysis. Additional data were collected via capstone interviews conducted specifically for this case study. Each data source is described below. Primary data collection and the use of secondary data for this case study were reviewed and approved by Cornell University’s internal review board, and prior consent was obtained from each capstone interviewee.

### Needs and Resources Assessment

Using a process from the Center for Community Health and Development (2018) and adapted by ACT, each program coordinator conducted a three-part local needs and resources assessment. In the *community brainstorm* component, community college and school district program coordinators generated a list of organizations that serve young parents in their community, noting those with whom they had or wanted to build relationships. Next, program coordinators conducted *key informant interviews* with representatives of six to eight entities that serve young parents. The purpose of these interviews was to better understand each organization’s programming, elicit informants’ perceptions of young parents’ needs, and gather information about the local system of supports for young parents. Coordinators used the list of service providers identified in the community brainstorm process to choose key informants, making their selection based on (1) the feasibility and desirability of developing a relationship with the key informant’s organization and (2) the informant’s anticipated level of knowledge regarding the needs of and resources available to young parents. Key informants included representatives of both community-based and district programs. Finally, *focus groups* were conducted with expectant and parenting young people in each community to better understand their needs, resources, sources of support, and barriers to obtaining support.

### Quarterly Reports

Across Pathways, each program coordinator reported quarterly on the funded organization’s progress implementing the initiative, including new and increasingly close connections to resource providers within the organization and the larger community, trainings they conducted, resource awareness-building efforts, work with individual expectant and parenting students, and descriptions of successes and challenges.

### Social Network Analysis

In years two and four of the four-year initiative, funded organizations used a social network analysis tool to map and describe their local network of community organizations and school-based programs serving young parents, characterizing relationships between all organizations in the network. Organizational relationships were described in terms of presence/absence, frequency of contact, level of collaboration, and value and trust within that relationship. This information was used to compute structural characteristics of the overall network, such as breadth, density, degree centralization, and trust (see Retrum et al. 2013 for an overview of these terms).

### Sustainability Plans

Each program coordinator documented strategies for continuing the work of Pathways beyond the funding cycle. The document containing these sustainability strategies also included the funded organizations’ results of the Program Sustainability Tool.^1^

### Capstone Interviews

At the end of the initiative, ACT staff conducted interviews with the district’s Pathways program coordinator and two other stakeholders whose family support program (FSP), discussed below, had emerged as essential to systems change within the district. Administrators from another such program, the school-based health centers (SBHC), were invited but unable to participate. Through these interviews, we sought to elicit perceptions of (1) the organizational and community context prior to Pathways; (2) changes resulting from Pathways efforts, including the initiative’s contributions, challenges, and promotion of collaboration among organizations; and (3) the sustainability of changes that benefit young families.

### Analysis

The research team, composed of members of NYSDOH and ACT, used the project planning and implementation data along with capstone interviews to conduct an intrinsic case study, an exploratory approach that focuses on deeply understanding the uniqueness of the case itself in order to describe its complexity and richness (Grandy 2010; Stake 2000). This approach contrasts with other types of case study analysis, which may focus on building theory or generalizing across cases (Grandy 2010). In this approach, data sources and analysis methods are qualitative, allowing participants and researchers to reconstruct the experience of the case by focusing more on interpreting meaning and less on generalizing findings (Grandy 2010; Patton 2002; Stake 2000).

All qualitative data sources were imported into the qualitative analysis software Dedoose (2018), a secure web-based application for organizing qualitative data and applying codes. Data were systematically reviewed by the research team to identify the existing context prior to the start of the initiative, the supports and other changes that resulted from the initiative, and the efforts to be maintained post initiative. We used an inductive approach to data analysis; research team members individually identified emergent themes and then, through discussion, developed the final set of codes. Two members of the research team then coded each data source. Discrepancies in codes were discussed until consensus was achieved. Following this content analysis, researchers identified main and sub-themes, discussed those triangulated by a variety of data sources, and developed the case study narrative.

Social network data were analyzed with the Program to Analyze, Record, and Track Networks to Enhance Relationships (PARTNER tool; Visible Network Labs 2010), an Excel-based macro to compute network and individual organization scores and create network maps. The PARTNER tool is described in more depth in the paper by Purington, Stupp, Welker, Powers, and Banikya-Leaseburg in this issue.

## Results

### Preexisting Context

Prior to Pathways, many programs and individuals worked to support young parents in this community. However, key programs worked in parallel rather than coordination, which limited their effectiveness.

Focus group respondents and several key informants spontaneously named “support” or “mental health” services as being an important yet largely unmet need for young parents. Services where young people felt welcomed and accepted were cited as the most helpful. The lack of support was often strongly felt and was raised several times during focus groups. Though family was a source of support for many, young parents reported depending on social services, family planning agencies, food pantries, and shelters, in addition to services at school, to meet their concrete needs.

Focus group participants also reported difficulty accessing existing services, often describing logistical factors that forced them to choose between competing priorities. One key informant summarized these challenges:

Many of the problems are larger systems problems…the services are inaccessible. It is routinely difficult to access services via phone. For example, [for] food stamps or public assistance, [a] teen is sent to multiple appointments in different locations with no [money for transportation] and during school hours, making it extremely challenging… Students miss school trying to attend pediatric appointments for their children…

According to focus group participants, key informants, and capstone interviewees, the most critical service providers for young parents within the school district were the FSP and SBHCs. Located in some high schools, FSPs provide free child care and early childhood education to children of parenting students to help parents stay on track toward graduation. These programs also support the transition into parenthood by providing counseling, academic guidance, and advocacy services. SBHCs, also located in school buildings, provide primary and mental health services to all students. One focus group participant enrolled in the FSP said that raising a child without FSP support would be impossible. Another participant said she chose to attend a particular high school because the FSP was available. Key informants reported that SBHCs can provide urgent health care and may be the first to identify pregnant students. Both entities provide essential services for expectant and parenting students.

However, findings from the key informants and capstone interviewees indicated that, prior to Pathways, programs in both high schools and the broader community worked in parallel rather than in coordination, which limited their utility. Social network analysis revealed that communication between network members was infrequent, and the network displayed low levels of cohesion and limited connection; collaboration was limited to networking and low-level cooperation, which includes some defined ways of working together but completely independent decision making (Frey et al. 2006; see Purington et al. in this issue for further discussion of the social network analysis).

Key informants and capstone interviewees reported that prior to Pathways, coordination between the FSPs and SBHCs was inconsistent. Though both FSPs and SBHCs serve expectant and parenting students within school buildings, the timing of their engagement with young parents is different. SBHCs typically identify students during pregnancy, and FSPs engage students when they return to school postpartum. The gap in young parent engagement between the start of pregnancy and exiting school for delivery and maternity leave was identified as an opportunity for coordination between the FSP and SBHCs. Some key informants reported that the application process required to access FSP services was not clearly understood by those who might refer young parents. As revealed in the focus groups, young parents did not understand how these two programs might connect. One key informant reported that the SBHC and FSP were physically located in the same hallway of one school but did not work together. The lack of coordination and communication was identified by sources as one of the largest barriers for expectant and parenting students accessing and utilizing important services in their school community, resulting in young parents being unaware of services intended to help them continue in school.

### Pathways to Success Activities

#### Facilitating Connections

Pathways became pivotal in facilitating connections and coordinating existing services for young parents in the district, with the Pathways program coordinator becoming a critical point of connection between organizations in the school and community. Drawing from the needs and resources assessment and early conversations with potential partners from other community organizations, the program coordinator recognized that although there appeared to be substantial support, “when you really start digging in and start meeting with people, they really aren’t set up for supporting the teens in school.” Appointment times are during school hours; long wait times force students to miss school; and collecting documentation is extremely difficult for young teens, many of whom end up missing days of school trying to manage public benefits.

Through meetings, phone calls, and other informal conversations, the program coordinator aimed to help school and community organizations become aware of the overlap in their missions and recognize the competing priorities of young student parents, an important step toward working more collaboratively. She regularly communicated with service providers about their resources and organizational processes (such as eligibility and referral requirements) and the barriers young parents encountered seeking assistance, including required paperwork and the need for multiple visits. These discussions helped identify areas for system improvement and facilitated discussion of solutions, such as providing transportation, clarifying paperwork requirements prior to appointments, and combining appointments when possible. The program coordinator became a repository of information about which services were offered, where, and who was eligible to receive them, and she shared this information with other resource providers whenever possible.

While working to build relationships between community-based organizations serving young parents, the program coordinator identified addressing the school district’s own organizational complexity and inefficiencies as a top priority; she believed internal relationships should be strengthened before focusing on external connections. Facilitating collaboration between the FSPs and SBHCs became a central focus. She began meeting with representatives of the FSPs and SBHCs, individually at first, and sharing knowledge of their programs’ goals and efforts with each other. As one capstone interviewee described, she was doing a lot of “myth debunking” and sharing accurate, useful information about services.

Next, the program coordinator worked to directly connect these key partners by convening joint meetings. One capstone interviewee shared that this was achieved by the FSP staff engaging in “quite a few conversations with [the program coordinator] to talk about the current state, what do relationships look like, where are there opportunities to strengthen, where are there challenges.” The FSP program staff and Pathways program coordinator then met with staff from SBHCs to streamline and resolve some of the challenges; they discovered that “much of the issues were from relationships between [FSP staff] and SBHC [and a lack of] understanding what is offered by each.” By facilitating relationships between the SBHCs and FSPs, the program coordinator intended to help programs provide expectant and parenting students in the school district with more seamless access to health services, academic and social/emotional support, childcare during school hours, and early childhood education.

#### Promoting Collaboration

Efforts to promote communication and coordination revealed another barrier to collaboration. One capstone interviewee reported that many staff members in the SBHCs and FSPs were “stuck in their silos” at the start of Pathways and not interested in talking with other programs about their work with students. Though the efforts of SBHCs and FSPs before Pathways were complementary, capstone interviewees expressed that their separate administrative objectives and oversight sometimes created friction, resulting in staff frustration. For example, SBHC and FSP policies prohibited sharing certain information between programs, making some staff members believe others were withholding information, as one capstone interviewee described.

To help address conflicts, the program coordinator convened a series of professional development team-building trainings held in three phases, each building on the previous one. The trainings focused on establishing a positive team culture and improving communication and trust among group members. SBHC and FSP staff learned about their respective services and resources and dispelled myths about their capacities, which helped them understand how their programs could better collaborate to holistically support the student parent and the child. Capstone interviewees reported that these trainings led to increased transparency and thus a willingness to collaborate across programs. Additionally, staff found great value in the opportunity to meet face to face and develop relationships.

As more collaborative relationships were developed, capstone interviewees reported it became easier to communicate, write referrals, and inform young parents of available services. Because of connections made as a result of Pathways, staff in certain SBHCs and FSPs began having daily phone calls with each other to discuss needed services for individuals. FSP program administrators reported that this dramatic increase in communication fostered greater coordination of efforts. According to these capstone interviewees, the Pathways program coordinator in the district played an enormous role in fostering these connections. Improved relationship quality between the FSPs and SBHCs has been one of the most successful changes resulting from Pathways.

### Sustainability

Much has been achieved through Pathways, and efforts to support young parents in this school district will continue beyond the grant cycle. Institutionalizing these changes through policies and procedures within an organization is one way to sustain impact (Wolff 2010). Dialogue between organizations that began during this grant cycle can be expected to continue after funding because clearly identified next steps to sustain and advance the accomplishments have been planned. For example, at the time of the capstone interviews, a “fast-tracking” process was in development and was a top priority across programs in the district. This process reduces the bureaucratic steps for young parents to access educational support services, to keep them engaged with school and help them graduate. The fast-tracking process is expected to expedite students’ path to graduation by helping them achieve their required academic credits more efficiently.

A similar approach to accessing services in the community would also support young student parents. One capstone interviewee reported, “A lot of [student parents] are already struggling academically, and then they miss a lot of school when they are trying to access public benefits. The systems are not set up to support the young people that are already slipping through the cracks.” During the needs and resources assessment, respondents suggested that streamlining this process would increase access to and use of resources. Pathways activities aimed at promoting collaboration and reducing institutional friction have led to increased buy-in from organizations and stakeholders. This buy-in is paramount to their commitment to continuing to develop the collaborative process, which, according to key stakeholders interviewed, has strengthened throughout the duration of Pathways.

Stakeholders expressed their intention to continue service coordination and mutual referrals between the SBHCs and the FSPs after Pathways. Self-assessments documented within the sustainability plan revealed this funded partner was very strong in developing a leadership team and securing community support, findings echoed in the capstone interviews. However, without the Pathways program coordinator to continue facilitating conversations, this degree of communication may be difficult to maintain.

## Discussion

Through an intrinsic case study approach, we sought to understand how the Pathways initiative enabled staff in one large school district to implement sustainable efforts to strengthen systems serving young parents. This case study illustrates how an approach that deeply explores existing systems can identify and address barriers to service awareness, access, and utilization, potentially suggesting strategies for communities looking to implement similar efforts.

Although communities often have many resources for young parents, bureaucratic policies and siloed system structures create barriers to access. The results of this case study paint a picture of disconnected services and missed opportunities prior to Pathways implementation and show that efforts to promote intra- and inter-organization communication, coordination, and—ultimately—collaboration may help reduce barriers to service. Having a dedicated program coordinator to help organizations recognize the overlap in their programs’ missions, facilitate efforts to ease conflict, and promote communication and collaboration can foster systems-level change to support young parents.

Though the findings from this case study are rich, they are limited by the data sources used and cannot be externally generalized. The research team sought to limit new primary data collection for the case study in order to limit the burden on study participants, instead drawing from existing data documented during Pathways planning and implementation; this approach limited the questions we could ask of these data. These sources were complemented by capstone interviews, which aimed to elicit a bigger picture perspective of the initiative and surrounding context, but these interviews were incomplete because not all invited respondents participated. As a result, findings were limited by the available data and respondent perspectives. Additionally, outcomes for individual young parents—the ultimate aim of the larger Pathways project—were not available data for this case study. Though the approach of systems-level change to improve individual outcomes is research based (Bloxham 1997; Rosell et al. 2010), this connection was not tested in this study. Rather, with this case study, we focused on deeply understanding the efforts required to bring about systems change. Future research should seek to connect systems-level change to individual outcomes and assess the sustainability of those changes.

Although this research is a case study of a single systems-change effort, the lessons learned may be relevant for communities seeking to do similar work. Understanding the organizational and community contexts into which a new initiative is being inserted is critical. Longstanding and unique ways organizations work (and do *not* work) together must be acknowledged and addressed. Increased coordination between service providers is possible by raising awareness of the work of similar organizations, identifying common goals, and providing opportunities for increased communication. By assessing barriers to collaboration—such as friction between individual members of organizations—it becomes possible to identify and implement useful strategies (i.e., training and team-building activities) to promote true collaboration. This study demonstrates that by developing relationships and instituting collaborative and responsive processes (such as routine communication and reducing bureaucratic barriers), a new way of working together on behalf of the priority population can be sustained.

## Footnotes

1 The Program Sustainability Assessment Tool addresses eight domains of sustainability and was created by the Center for Public Health Systems Science at Washington University in St. Louis. See https://sustaintool.org/psat/assess/#about-assessment.
